# Event and Comment

**Published:** 1922-01

**Authors:** 


### DENTAL REGISTER

THE MAGAZINE OF DENTISTRY

Vol. LXXVI JANUARY, 1922 No. 1

### EVENT AND COMMENT

**What will the New Year do for Dentistry?** Without reciting the accomplishments of the past year we are always anxious to know what we are to expect for the next year. We are now up to this move, and in some respects we are hoping it will bring many desirable conditions we failed to realize last year. There are many things we expected to do that we either forgot or neglected to get done last year, and we confidently hope to do much better this year than we have accomplished in any year previously.

The war is over, but it has left a lot of trouble aside from the financial burdens we shall need to carry for many years to come. But we still have hope, and we have strength and will to do some things that we think desirable and possible, and we shall tackle the new year with all the power we have, and it may be that new aspirations will come to cheer us to the place where the best that has ever come to us will reward our efforts. Hope is certainly one of the grandest possessions we have, and with her help and the traditions of past achievements we shall assuredly do things to cheer and accomplish some of the aspirations we have long desired.

Is the problem of Oral Hygiene in safe hands; and can we hope for preventive dentistry? Are we doing our full duty as a profession to relieve suffering humanity with unselfish ministrations?

Is there any progress in our endeavor to prevent dental caries and peridental diseases?

Have we come to a satisfactory conclusion as to how we can handle crown and bridge cases without endangering patients' health, and at prohibitive expense to poor people?

Is the partial denture as well cared for as the full denture has been; and do patients yet know how important it is that all artificial dentures should be made more wholesome?

How can we increase the efficiency of instruction in dental education without reducing the number of students and thus reduce the number of needed practitioners?

These are some new and needed problems to be solved this year. Can they be done and we maintain our repu-
this year. Can these be done and we maintain our reputation for progressive and safe dentistry?

